# Bears and berries: species-specific selective foraging on a patchily distributed food resource in a human-altered landscape

**DOI:** 10.1007/s00265-016-2106-2

**Published:** 2016-03-31

**Authors:** Anne G. Hertel, Sam M. J. G. Steyaert, Andreas Zedrosser, Atle Mysterud, Hanna K. Lodberg-Holm, Henriette Wathne Gelink, Jonas Kindberg, Jon E. Swenson

**Affiliations:** Department of Ecology and Natural Resource Management, Norwegian University of Life Sciences, 1430 Ås, Norway; Department of Environmental and Health Sciences, Telemark University College, 3901 Porsgrunn, Norway; Institute for Wildlife Biology and Game Management, University for Natural Resources and Life Sciences, 1180 Vienna, Austria; Department of Biosciences, Centre for Ecological and Evolutionary Synthesis (CEES), University of Oslo, 0316 Oslo, Norway; Swedish University of Agricultural Sciences, 90183 Umeå, Sweden; Norwegian Institute for Nature Research, 7485 Trondheim, Norway

**Keywords:** Bilberry, Brown bear, Lingonberry, Movement trajectories, Optimal foraging, Sugar content

## Abstract

**Abstract:**

When animals are faced with extraordinary energy-consuming events, like hibernation, finding abundant, energy-rich food resources becomes particularly important. The profitability of food resources can vary spatially, depending on occurrence, quality, and local abundance. Here, we used the brown bear (*Ursus arctos*) as a model species to quantify selective foraging on berries in different habitats during hyperphagia in autumn prior to hibernation. During the peak berry season in August and September, we sampled berry occurrence, abundance, and sugar content, a proxy for quality, at locations selected by bears for foraging and at random locations in the landscape. The factors determining selection of berries were species specific across the different habitats. Compared to random locations, bears selected locations with a higher probability of occurrence and higher abundance of bilberries (*Vaccinium myrtillus*) and a higher probability of occurrence, but not abundance, of lingonberries (*Vaccinium vitis-idaea*). Crowberries (*Empetrum hermaphroditum*) were least available and least used. Sugar content affected the selection of lingonberries, but not of bilberries. Abundance of bilberries at random locations decreased and abundance of lingonberries increased during fall, but bears did not adjust their foraging strategy by increasing selection for lingonberries. Forestry practices had a large effect on berry occurrence and abundance, and brown bears responded by foraging most selectively in mature forests and on clearcuts. This study shows that bears are successful in navigating human-shaped forest landscapes by using areas of higher than average berry abundance in a period when abundant food intake is particularly important to increase body mass prior to hibernation.

**Significance statement:**

Food resources heterogeneity, caused by spatial and temporal variation of specific foods, poses a challenge to foragers, particularly when faced with extraordinary energy-demanding events, like hibernation. Brown bears in Sweden inhabit a landscape shaped by forestry practices. Bilberries and lingonberries, the bears’ main food resources in autumn prior to hibernation, show different temporal and habitat-specific ripening patterns. We quantified the bears’ selective foraging on these berry species on clearcuts, bogs, young, and mature forests compared to random locations. Despite a temporal decline of ripe bilberries, bears used locations with a greater occurrence and abundance of bilberries, but not lingonberries. We conclude that bears successfully navigated in this heavily human-shaped landscape by selectively foraging in high-return habitats for bilberries, but did not compensate for the decline in bilberries by eating more lingonberries.

**Electronic supplementary material:**

The online version of this article (doi:10.1007/s00265-016-2106-2) contains supplementary material, which is available to authorized users.

## Introduction

Foraging efficiently in northern environments is challenging, due to patchily distributed forage and seasonal variability in resource quality and availability (Wiens 1976; Belovsky et al. 1989). Foraging theory predicts that animals strive to increase energy or nutrient intake per unit time, while also limiting searching costs (Emlen 1966; MacArthur and Pianka 1966), under constraints set by intrinsic (e.g., nutritional demand, sex, digestive physiology) and external factors (e.g., predation risk, thermal factors) (Belovsky 1981; Belovsky and Schmitz 1993; Nathan et al. 2008). Spatial and temporal scale is inherent in all foraging theory, both due to food distribution and the decisions made by the animals (Senft et al. 1987). Patchiness of forage is defined by (at least) a two-step hierarchy, first by general occurrence and second by abundance (Kotliar and Wiens 1990). The profitability of a food resource thus depends on its spatial occurrence (affecting search time), its quality in terms of energy and/or nutrient content, and its local abundance. For generalists, the challenge becomes to track the abundance and quality of different food items, which are rarely static over time, due to both seasonal changes and depletion due to use (Charnov 1976). Profitability of different food types may thus change over time, if their relative abundance changes through differential seasonality of foods (Hambäck 1998), and space, when habitat suitability for the occurrence of foods is altered naturally or anthropogenically (Nielsen et al. 2004). Hibernators like the brown bear (*Ursus arctos*) are faced with a particular need to maximize food intake preceding hibernation (Manchi and Swenson 2005; Robbins et al. 2012), because during winter, they are solely sustained by fat and lean reserves acquired during autumn (Nelson et al. 1983; Farley and Robbins 1995). Hibernators should thus be particularly responsive to environmental stochasticity and phenological changes in food availability when accumulating fat resources during hyperphagia (Ozgul et al. 2010; Tafani et al. 2013).

Bears increase their daily energetic intake substantially during fall (Nelson et al. 1983). They forage up to 14 h per day (Stelmock and Dean 1986) and increase their body mass in autumn by approximately 35 and 65 % for males and females, respectively, compared to their spring mass (Swenson et al. 2007). Brown bears are generalist omnivores, with populations in temperate boreal forests being more frugivorous than populations at lower latitudes (Bojarska and Selva 2012). At northern latitudes, berries are the most important forage for bears during fall. Berries are low in protein and high in carbohydrates, which can be easily converted into fatty tissue and are therefore an excellent food resource to increase body mass prior to hibernation (Eriksson and Ehrlén 1991; McDonald 2002). Even populations with access to salmon (*Oncorhynchus* sp.), a food source high in protein and offering three to four times greater energy intake per time unit than fruit (Welch et al. 1997), will readily forage on fruits (Fortin et al. 2007). This is likely because a diet consisting only of protein-rich meat/salmon or only of fruits entails high metabolic costs and potential nutritive deficits (Rode and Robbins 2000), thus setting a physiological constraint to pure energy maximization. Compared to berries or meat, herbaceous material should play a limited role as a food resource during hyperphagia, because its lower nutritional value makes it less suitable for rapid mass gain (Rode et al. 2001).

In Sweden, brown bears forage almost exclusively on bilberries (*Vaccinium myrtillus*), lingonberries (*Vaccinium vitis-idaea*), and crowberries (*Empetrum* spp.) from mid-July until den entry. Berries comprise approximately 44 % of the annual digestible energy intake and 68 % of the autumn digestible energy intake (Dahle et al. 1998; Persson et al. 2001; Stenset et al. 2016). Forbs, gramnoids, fungi, insects, and animal matter are consumed in negligible quantities at this time of the year (Stenset et al. 2016). Bilberry is the most common berry species in Sweden, and its plants cover up to 17 % of the forest floor in the Swedish boreal forests (Kardell 1979). In comparison, lingonberries cover only about 5 % of the forest floor (Kardell 1979). Crowberries are important bear foods in northern Scandinavia (Persson et al. 2001), but are less common in the landscapes of central and southern Scandinavia (Kardell and Eriksson 2011).

Forest management affects the occurrence of berry plants and the abundance of berries (Kardell 1979, 1980). Open, mature forests with intermediate light conditions offer the best growing conditions for bilberries and good conditions for lingonberries. Clearcutting decreases bilberry cover, because plants are destroyed during the harvesting and replantation process, although it creates growing conditions that are optimal for lingonberry (Kardell 1979; Atlegrim and Sjöberg 1996). Kardell and Eriksson (2011) reported that direct sunlight incidence increases fertility on early clearcuts for both bilberry and lingonberry, leading to high local berry abundances. Both bilberry and lingonberry plants are outcompeted by pioneer shrubs and trees when the forest starts to close (Kardell 1979; Kardell and Eriksson 2011).

Bears are expected to select for berries with a high dry-matter digestibility and a high carbohydrate/sugar content to optimize mass gain. Because the distribution of berries is highly variable both temporally and spatially, the availability and distribution of the different berry species might be equally important to energy content itself in affecting food choice (McLellan and Hovey 1995). Thus, foraging on different berry species by bears should be determined by a combination of availability, search time, and food quality (Krebs and McCleery 1984).

Here, we aim to quantify whether Scandinavian brown bears selectively use locations of higher berry abundance than random locations and we test whether bears selected more strongly for high abundance locations of one of the three available berry species during the critical period of hyperphagia before hibernation. Additionally, we measured sugar content of berries as an indicator of nutritional quality. We compared whether berries at foraging locations (derived from GPS relocation data) were energetically more valuable than berries at random locations. We hypothesized (H1) that bears selected for locations with a higher probability of berry occurrence and higher berry abundance in comparison to random locations, and (H2) that bears select bilberries over crow- and lingonberries, as reported in earlier studies (Stenset et al. 2016), because they are most commonly available (Kardell and Eriksson 2011) and have the highest dry-matter digestibility (Welch et al. 1997). However, we expected that bears would respond to temporal variations in the relative availability of berry species during the autumn and hypothesized (H3) that bears used and selected locations of the berry species that were most abundant at a given time. In addition, we hypothesized (H4) that land-use practices affecting local habitat characteristics were determinants of berry occurrence and abundance, with mature forests and clearcuts being more important berry foraging habitats, compared to young forests or bogs. Lastly (H5), we expected that bears used berries of better quality (higher sugar content) than randomly available to maximize mass gain.

## Materials and methods

### Study area and study species

The study area was situated in south-central Sweden in the counties of Dalarna and Gӓvleborg (online resource 1). The terrain is rolling, with elevations between 250 and 650 m above sea level and mostly covered by commercial coniferous forests dominated by Scots pine (*Pinus sylvestris*) and Norway spruce (*Picea abies*). Timber harvesting rotation time is about 100 years (Linder and Östlund 1998). Approximately 8 % of the area is recently logged forest (clearcuts, 0–10 years old), and 42 % of the forest stands are younger than 35 years (Swenson et al. 1999). Brown bear population density is approximately 30 individuals/1000 km^2^ (Bellemain et al. 2005). Within the long-term Scandinavian Brown Bear Research Project (SBBRP), 40–50 bears are equipped annually with GPS-GSM collars (Vectronic Aerospace GmBh, Berlin, Germany). See Arnemo and Fahlman (2011) for details on capture and handling. All animal capture and handling was approved by the Ethical Committee on Animal Experiments in Uppsala, Sweden and the Swedish Environmental Protection Agency.

### Identifying foraging behavior from GPS trajectories

We monitored seven GPS-collared bears (four males, three females) between 7 August and 4 September 2014 (online resource 2). The GPS collars were scheduled to record a GPS fix every 30 min (48 positions/day). Spatial accuracy of the GPS collars was approximately 10 m (Moe et al. 2007; Arnemo and Fahlman 2011). We used the R package (R Development Core Team 2013) adehabitatLT (Calenge 2006) to analyze bear movement trajectories. We calculated movement distances as the Euclidean distance between two successive locations. Low satellite coverage may lead to failed GPS fixes (Moe et al. 2007). To avoid false distance calculations, we set all missing locations to NA. This caused missing distance calculations for the 30-min period before and after the missing event and avoided falsely including these positions in a foraging trajectory.

Movement trajectory and cluster analysis is a common technique for identifying behavior, such as resting (Ordiz et al. 2011), predation (Rauset et al. 2012), and foraging (Bastille-Rousseau et al. 2011). The main challenge to identify foraging on vegetation from GPS tracking data is long intervals between GPS fixes (Cristescu et al. 2015). Because berry foraging by bears is characterized by slow and meandering movements (Stelmock and Dean 1986; Welch et al. 1997), we considered trajectories of at least three consecutive fixes (i.e., 1.5 h) with a minimum movement distance of 25 m and a maximum of 300 m as berry foraging behavior. We downloaded data daily to identify foraging trajectories. To avoid spatial autocorrelation in berry abundance between sample points, we only sampled berry abundance once, at the second position of each trajectory. If trajectories were large, containing ≥7 fixes in a row (>3.5 h of consistently slow movements), we also sampled the second to the last position, because bears had moved for 2 h and we therefore considered the autocorrelation to be low. We excluded all foraging trajectories within a 200-m buffer around known slaughter dump sites and agricultural fields (*Avena sativa*), because bears occasionally forage on slaughter remains from pigs (*Sus scrofa*) or hunter-killed moose (*Alces alces*) or on agricultural crops during autumn (Elfström et al. 2014).

### Berry plot inventories

Berry foraging plots (from here on referred to as berry plots) were identified in the field using a hand-held GPS with an accuracy of approximately 10 m (Moe et al. 2007). From the zero position, we randomly selected a berry plot location by walking 0–9 m (depending on the last number of the plot’s *Y* coordinate) in a randomly assigned direction (North, East, South, West, depending on the last digit of the plot’s *X* coordinate). This location marked the center of a sample quadrate of 1 m^2^. If a selected plot contained obvious signs of foraging, we relocated the sample plot to the opposite direction from the original GPS location, but at the same distance. We defined foraging signs as stripped and bent twigs and fallen berries and/or leaves (Welch et al. 1997). We counted the number of ripe berries within the sample quadrate. We considered berries to be ripe when it was possible to squeeze them between two fingers with relative ease, and more than half of the berries’ coloration had changed from green to blue or red. For each plot, we determined the sugar content in the juice of a random subsample of five ripe berries. Total soluble solids (TSS), which is the combination of sucrose, fructose, glucose, were measured in %Brix (percentage of sugar in an aqueous solution; 1 %Brix ≌ 1 g sucrose in 100 g sucrose water solution) using a digital wine refractometer MA885 (Milwaukee Instruments, Inc., Rocky Mount, NC, USA). We calibrated the refractometer with distilled water. Berries were homogenized and the juice was filtrated through gauze before applied to the prism. The refractometer measures how light passes through the solution as a refractive index. The index is used to calculate the physical properties of the solution. We classified the habitat surrounding a plot location into mature forest (average tree height >10 m), young forest (tree height >1.3 and <10 m), clearcut (mostly bare soil with trees <1.3 m), and bog (non- or sparsely forested areas with wet soils), based on the Swedish National Forestry Inventory (Esseen et al. 2008). We excluded locations in all other habitat types (*n* = 20) from the analysis. We recorded the ordinal day we described the plot. Positions were sampled 1 to 19 (median 3, 1st quartile and 3rd quartile: 2 and 5) days after the position had been recorded. We compared berry occurrence (present or absent) and abundance (number of berries) in berry plots located at bear foraging positions with berry plots located at 375 randomly selected locations in the study area encompassing the bears’ home ranges during the same time period. All plot inventories followed the same procedure. It was not possible to have a blinded sampling of random and bear positions as bears had known home ranges and additional signs of bear presence were recorded in the surrounding of foraging locations but not of random locations.

### GIS-derived predictor variables

To control for terrain characteristics affecting berry occurrence and abundance, we extracted elevation (200–600 m), aspect (N, E, S, W), and slope (0–77°) from a 2 × 2-m digital elevation model (Lantmäteriet, license no. i2014/764). We also calculated the normalized difference vegetation index (NDVI; −1–0.86) from Resourcesat satellite imagery obtained during the sampling season (grid size 23.5 m). NDVI is a proxy for plant vegetation biomass and thus an index of vegetation density (Myneni et al. 1997; Pettorelli et al. 2005). NDVI should be negatively correlated with berry abundance, because berry shrubs grow best in open forests and are outcompeted at sites of high vegetation density (Kardell 1979).

### Statistical analyses

We analyzed the number of ripe bilberries, lingonberries, and crowberries at a given site separately. We inspected predictor variables for collinearity and used variance inflation factor (VIF) with a cut-off value of 3 to decide which predictors to drop from the analyses (Zuur et al. 2009). Because our berry count data were zero-inflated and overdispersed, we modeled berry occurrence and abundance separately with negative-binomial hurdle models (Zeileis et al. 2008). Hurdle models treat the data in two separate ways. First, a binomial model identifies which factors affect the occurrence of berries at a given site (value 1 for all plots with 1 or more ripe berries) compared to plots with no berry occurrence (i.e., plots with zero berries). Second, a zero-truncated negative binomial model identifies which factors influence the abundance of berries, once they are present in a plot (value of berry count for all plots with 1 or more counts) (Ridout et al. 1998; Potts and Elith 2006). The underlying assumption of these models is that zero observations are true negatives (e.g., not due to sampling or observer error). As we based our foraging plot selection on seven monitored bears, bear ID was included as a random effect. We assigned random plots randomly to one of the bear IDs to fit a mixed-effects model. We used the glmmADMB package (Fournier et al. 2012; Skaug et al. 2013) to fit the zero-truncated part of the mixed-effects hurdle model and the lme4 package (Bates et al. 2014) to fit the binomial part. We based model selection on a stepwise backward model selection, removing nonsignificant covariates or interactions one at a time and comparing full and reduced models with likelihood ratio tests (LRs). Both parts of the model started with a full model, containing plot type (random vs. bear foraging), landscape characteristics, habitat, and sampling date as main effects, an interaction term between habitat and plot type, and an interaction term between date and plot type. Inspection of diagnostic plots did not reveal any nonlinear pattern for sampling date. We used *α* = 0.05 as a threshold for statistical significance in all analyses. All graphical displays were produced for predicted population mean effects and are thus based on the optimal model, but fitted as a binomial or negative binomial GLM, instead of the GLMM. Post hoc pairwise comparisons were computed for multilevel factors and interactions using the glht() function of the multcomp package (Hothorn et al. 2008).

We tested for a temporal shift in the abundance of bilberries and lingonberries at foraging locations only. Based on our results for berry occurrence and abundance, we focused our analysis on the two most suitable berry habitat types, mature forest and clearcuts. We modeled the number of berries as a function of the main fixed effects; berry species, sampling date, and habitat. As we were interested in quantifying how the abundance of ripe berries at foraging locations changes between species, both over time and between habitats, we modeled these effects as a three-way interaction. We also included two-way interactions to determine whether the number of berries per species varied among the habitats or over time. As in the previous models, the variation in berry abundance was greater than its mean; hence, count data were overdispersed, so we chose a negative binomial distribution (Zuur et al. 2009). Because we sampled bilberries and lingonberries in the same plot locations, we added plot ID as a random effect. Computations were carried out with the R package glmmADMB (Skaug et al. 2013).

To assess whether bears selected for berries with a higher sugar content, we modeled TSS in bilberries (*n* = 334) and lingonberries (*n* = 265) as a function of the full set of predictor variables (habitat, sampling date, aspect, slope, elevation, NDVI, and plot type) in a linear mixed-effects model (Pinheiro et al. 2013) with bear ID as a random intercept. We applied backwards model selection and likelihood ratio tests (see above) to find the most parsimonious model explaining sugar content in berries.

## Results

GPS collar fix success was 92 ± 7 % (mean ± SE) for all bears combined, resulting in few missing distance calculations (online resource 2). We identified 395 foraging trajectories that contained on average 5.28 (range 3–18) consecutive GPS fixes. After excluding other habitats and plots with missing values for either of the covariates, 346 bear foraging and 373 random plots were included in the statistical analyses (online resource 1). We found signs of foraging in the immediate surroundings of 70 % of the foraging plots. Overall, 82 % of the foraging plots contained ripe bilberries, 54 % contained ripe lingonberries, and 15 % contained neither ripe bilberries nor ripe lingonberries. The numbers of ripe bilberries and ripe lingonberries in a plot were not correlated (*r* = 0.021, *p* = 0.582). Ripe crowberries occurred in only 10 % of the sampled plots (foraging plots *n* = 26, random plots *n* = 45), with generally very low abundances (less than six berries in 50 % of plots with crowberry occurrence) and few plots with high abundances (up to 159). For the crowberry data, zero inflation was too high and variation too large to obtain reliable statistical estimates for our research questions, so these data were not analyzed.

The average fresh weight of ripe berries was 0.3 g for bilberries and 0.23 g for lingon- and crowberries (Table 1). Berry density (average number of berries per m^2^ plant coverage calculated from all locations where berries were present) was 49 berries/m^2^ for crowberry, 75 berries/m^2^ for bilberry, and 102 berries/m^2^ for lingonberry. Bilberry shrubs were tallest and crowberry shrubs lowest (Table 1).

**Table 1:** Nutritional and presentational properties of the three most common berry species in Sweden

	Bilberry (*V. myrtillus*)	Lingonberry (*V. vitis-idaea*)	Crowberry (*Empetrum* sp.)
Weight (g/berry)	0.30 ± 0.09	0.23 ± 0.06	0.23 ± 0.04
Density (no. of berries/m^2^ cover)	75 ± 77	102 ± 174	49 ± 75
TSS (%Brix)	8.48 ± 1.42	12.04 ± 1.44	5.57 ± 0.9
Shrub height	17.49 ± 10.67	10.06 ± 6.78	2.25 ± 5.65
Dry matter digestibility (%)	72.2^a^	70^a^	49.2^a^
Metabolizable energy (kcal/g)			
Sum	2.74^b^	2.82^b^	2.09^b^
Protein	0.16^b^	0.15^b^	0.12^b^
Carbohydrate	2.34^b^	2.4^b^	1.53^b^
Lipid	0.24^b^	0.28^b^	0.44^b^

The first four columns refer to the results from our study

^a^Welch et al. (1997)

^b^Coogan et al. (2014)

### Bilberry occurrence

**Fig 1:**
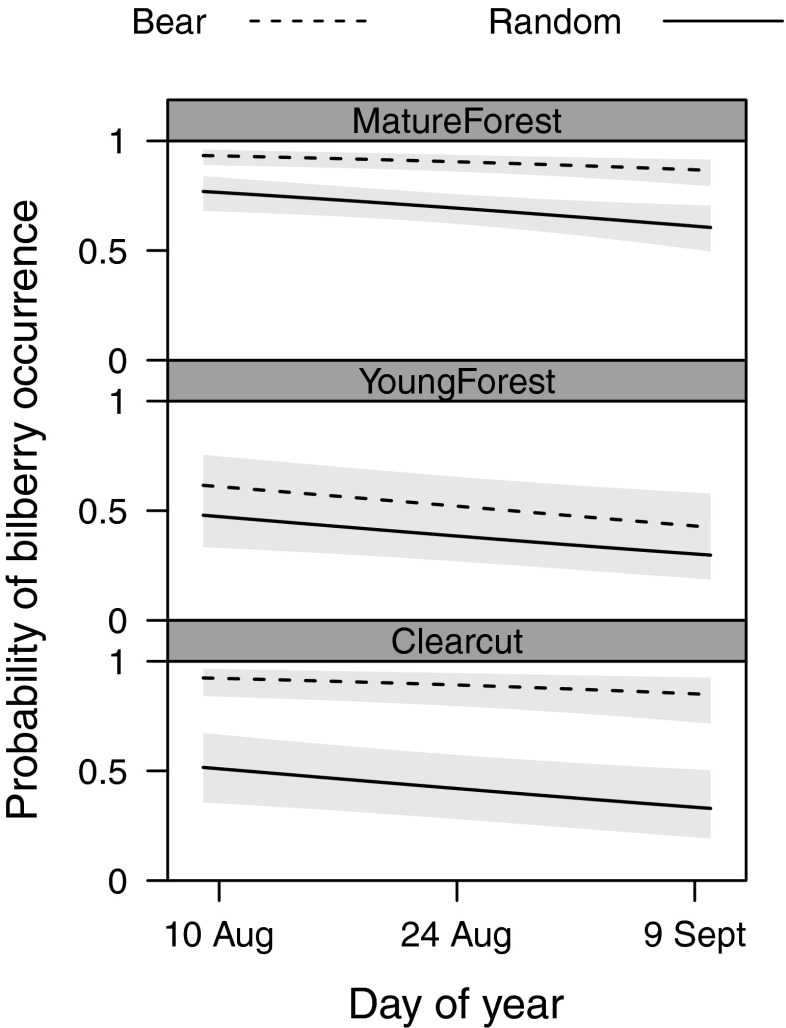
Probability of bilberry occurrence at random GPS positions (*solid line*) and locations selected by brown bears (*dashed line*) along a 32-day gradient in south-central Sweden, while keeping elevation constant at the mean elevation value. *Gray areas* represent the 95 % confidence intervals around the estimates. Contrasts are shown for young forest, mature forest, and clearcuts. Predictions are based on population-level means

Bilberry occurrence was significantly affected by an interaction between plot type (foraging vs. random) and habitat (LR *χ*^2^ = 9.935, *df* = 3, *p* = 0.019). A post hoc multiple comparisons of means showed that, in random plots, mature forest had a higher probability of bilberry occurrence than any other habitat (Table 2). Comparing bear foraging and random plots, probability of bilberry occurrence was around 90 % in foraging plots in mature forests and clearcuts (Fig. 1), which was significantly higher than the probability of occurrence in random plots of both habitat types. Probability of occurrence did not differ between foraging and random plots on bogs or young forests. Further, foraging plots on clearcuts and in mature forests had a higher probability of berry occurrence than foraging plots in young forests (Fig. 1, Table 2). Probability of bilberry occurrence increased with elevation and decreased as the season progressed (Table 3). We did not find a significant interaction between sampling date and plot type (LR *χ*^2^ = 0.307, *df* = 1, *p* = 0.58), which indicated that bear selectivity did not change over the sampling season.

**Table 2:** Contrasts comparing the probability of bilberry occurrence in brown bear foraging plots and random plots and four habitat types in south-central Sweden, 2014

Multiple comparisons of means	*β* ± SE	*p*
Bear × Bog - Random × Bog	1.818 ± 0.788	0.267
Bear × Clearcut - Random × Clearcut	2.484 ± 0.507	<0.001
Bear × MatureForest - Random × MatureForest	1.47 ± 0.283	<0.001
Bear × YoungForest - Random × YoungForest	0.545 ± 0.398	0.858
Bear × Bog - Bear × YoungForest	−0.04 ± 0.779	1
Bear × Clearcut - Bear × Bog	2.135 ± 0.819	0.139
Bear × Clearcut - Bear × YoungForest	2.095 ± 0.485	<0.001
Bear × MatureForest - Bear × Bog	2.242 ± 0.754	0.053
Bear × MatureForest - Bear × Clearcut	0.107 ± 0.441	1
Bear × MatureForest - Bear × YoungForest	2.202 ± 0.367	<0.001
Random × Bog - Random × YoungForest	−1.313 ± 0.408	0.025
Random × Clearcut - Random × Bog	1.469 ± 0.443	0.018
Random × Clearcut - Random × YoungForest	0.156 ± 0.419	1
Random × MatureForest - Random × Bog	2.59 ± 0.348	<0.001
Random × MatureForest - Random × Clearcut	1.121 ± 0.36	0.034
Random × MatureForest - Random × YoungForest	1.277 ± 0.317	0.001

Post hoc multiple comparisons of means were based on the significant interaction term between plot type and habitat in the optimal model explaining berry occurrence

*p* values were single-step adjusted for multiple testing

**Table 3:** Model estimates, standard errors, and *p* values for variables describing occurrence and abundance of bilberries (BBs) and lingonberries (LBs) in random plots and plots used by foraging brown bears in south-central Sweden in the autumn of 2014

Explanatory variables	BB occurrence		BB abundance		LB occurrence		LB abundance	
	*β* ± SE	*p*	*β* ± SE	*p*	*β* ± SE	*p*	*β* ± SE	*p*
Plottype (Random = 0, Bear = 1)	2.297 ± 2.391	0.021	0.828 ± 0.09	<0.001	0.719 ± 0.175	<0.001		
Habitat (Bog = 0)								
Clearcut	1.469 ± 0.444	0.001	0.637 ± 0.241	0.008	2.706 ± 0.126	<0.001	0.990 ± 0.319	0.002
Mature Forest	2.59 ± 0.348	<0.001	0.574 ± 0.228	0.012	1.466 ± 0.359	<0.001	0.254 ± 0.297	0.392
Young Forest	1.313 ± 0.407	0.001	0.575 ± 0.253	0.023	1.644 ± 0.396	<0.001	0.637 ± 0.313	0.042
Elevation	0.003 ± 0.001	0.002	0.004 ± 0.001	0.010				
NDVI							−2.304 ± 0.893	0.01
Ordinal day	−0.022 ± 0.01	0.016	−0.008 ± 0.004	0.032	0.07 ± 0.009	<0.001	0.028 ± 0.006	<0.001
Plottype × Habitat (Random = 0; Bog = 0)								
Bear × Clearcut	0.665 ± 0.932	0.476						
Bear × Mature Forest	−0.349 ± 0.827	0.673						
Bear × Young Forest	−1.275 ± 0.879	0.147						

Zero hurdle model coefficients (occurrence) are based on binomial distributions with logit link. Count model coefficients (abundance) are based on a zero-truncated negative binomial distribution with log link

*NDVI* normalized difference vegetation index

### Bilberry abundance

**Fig 2:**
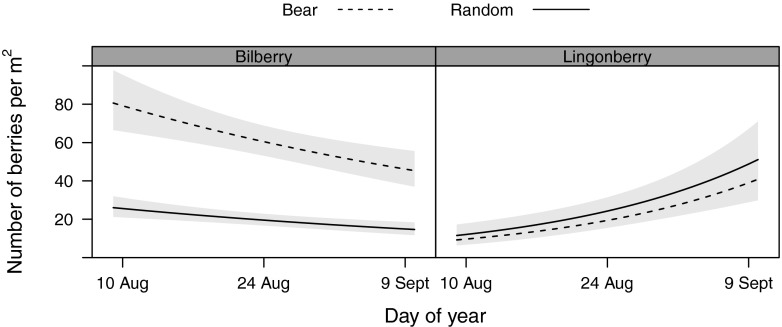
Predicted bilberry (*left*) and lingonberry (*right*) abundance per square meter on brown bear foraging plots and random plots in mature forests in south-central Sweden, displayed for a gradient of 32 days between 8 August and 4 September 2014. Estimates and their 95 % confidence interval are shown for random (*solid line*) and bear foraging plots (*dashed line*). Predictions are based on population-level means

The number of bilberries per square meter was significantly and up to three times higher in bear foraging plots than in random plots (Table 3, Fig. 2). Habitat significantly affected abundance (LR *χ*^2^ = 12.954, *df* = 3, *p* = 0.005). Post hoc tests revealed that bilberries were less abundant on bogs than on clearcuts (est ± SE 0.637 ± 0.241, *p* = 0.038). Bilberry abundance per square meter decreased with advancing season (Table 3), and by the beginning of September, bilberry numbers had dropped to half of that observed at the beginning of August (Fig. 2).

### Lingonberry occurrence

Bear foraging plots had a higher probability of lingonberry occurrence than random plots. The probability of ripe lingonberry occurrence varied among habitat types (LR *χ*^2^ = 56.026, *df* = 3, *p* < 0.001) and was highest on clearcuts and lowest on bogs (*p* < 0.05 for all pairwise comparisons). The probability of ripe berries occurring in a plot increased strongly with progressing season (Table 3).

### Lingonberry abundance

We did not find evidence that bears selected for areas of greater abundance of ripe lingonberries than found in random plots (Fig. 2). Berry abundance differed among habitat types (LR *χ*^2^ = 27.32, *df* = 3, *p* < 0.001). Post hoc multiple comparisons of means showed that lingonberry abundance was higher on clearcuts than on bogs (est ± SE = 0.990 ± 0.319, *p* = 0.01). Lingonberry abundance decreased with increasing NDVI and increased strongly with progression of the season (Table 3). Predicted numbers of ripe lingonberries were three times higher at the end of the sampling season than at the beginning.

### Seasonal shift in berry abundance by species at foraging locations

**Fig 3:**
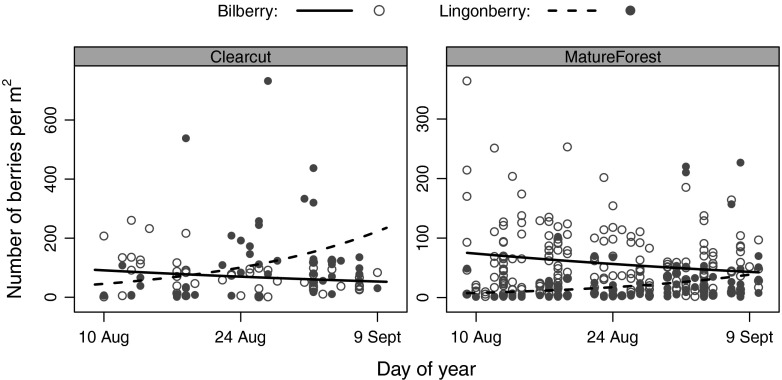
Observed and predicted numbers of bilberries and lingonberries in brown bear foraging plots along a 32-day gradient in south-central Sweden between 8 August and 4 September 2014. Observed abundances are shown as *filled circles* for lingonberries and *open circles* for bilberries. Predicted abundances are shown as *lines*. Estimates are shown for clearcuts and mature forest. Visualization of the results was based on predicted population-mean effects

Abundance of ripe berries in foraging plots was affected by an interaction between sampling date and berry species (LR *χ*^2^ = 33.56, *df* = 1, *p* < 0.001) and an interaction between habitat type and berry species (LR *χ*^2^ = 42.88, *df* = 1, *p* < 0.001), while controlling for the random effect of sampling plot (Table 4, Fig. 3). The number of ripe bilberries in mature forests exceeded the number of ripe lingonberries (post hoc pairwise comparisons est ± SE = 1.402 ± 0.205, *p* < 0.001), but there was no significant difference in the number of ripe bilberries and lingonberries on clearcuts (post hoc pairwise comparisons est ± SE = 0.402 ± 0.205, *p* = 0.183). Numbers of ripe bilberries decreased in both habitats over time, whereas numbers of ripe lingonberries increased (Fig. 3). Total number of berries in the two habitats did not differ over time, suggesting that a decrease in the availability of one was balanced by an increase in the number of the other species, keeping total berry abundance stable over time.

**Table 4:** Model estimates, standard errors, and *p* values for variables describing berry abundance in brown bear foraging plots in south-central Sweden in 2014

Explanatory variables	*β* ± SE	*p*
Species (bilberry = 0, lingonberry = 1)	−16.901 ± 2.896	<0.001
Ordinal day	−0.016 ± 0.007	0.022
Habitat (Clearcut = 0, MatureForest = 1)	−0.257 ± 0.158	0.105
Species × Ordinal day	0.072 ± 0.012	<0.001
Species × Habitat	−1.578 ± 0.233	<0.001

Coefficients are based on a zero-truncated negative binomial mixed-effects model controlling for sampling plot

### Sugar content

Bilberries selected by foraging brown bears did not differ in their sugar content from bilberries at random locations. Sugar content in bilberries was affected by terrain variables, NDVI, and sampling date (Table 5). Sugar content decreased with increasing NDVI, elevation, and as the season progressed. Post hoc multiple comparisons of means showed that berries on clearcuts contained more sugar than berries in mature forests (est ± SE = −0.672 ± 0.245, *p* = 0.016); no other pairwise comparison was significant. Bears selected lingonberries with a higher sugar content than lingonberries at random locations (Table 5). Lingonberry sugar content increased as the season progressed and decreased with increasing NDVI. Mean TSS of bilberries was significantly lower than mean TSS for lingonberries (*t* test *t* = 29.817, *p* < 0.001; Table 1). Mean TSS of crowberry was lower than for either bilberry (*t* test *t* = 21.436, *p* < 0.001) or lingonberry (*t* test *t* = 45.434, *p* < 0.001).

**Table 5:** Model estimates, standard errors, and *p* values for variables describing bilberry (BB) and lingonberry (LB) sugar content (TSS) in south-central Sweden in 2014

Explanatory variables	BB sugar		LB sugar	
	*β* ± SE	*p*	*β* ± SE	*p*
Plottype (Random = 0, Bear = 1)			0.579 ± 0.178	0.001
Habitat (Clearcut = 0)				
Mature Forest	−0.672 ± 0.249	0.007		
Young Forest	−0.477 ± 0.323	0.14		
Aspect (North = 0)				
East	0.384 ± 0.202	0.058		
South	0.565 ± 0.202	0.006		
West	0.449 ± 0.202	0.027		
Elevation	−0.003 ± 0.001	0.002		
NDVI	−5.903 ± 1.792	0.001	−4.439 ± 1.316	0.001
Ordinal day	−0.049 ± 0.008	<0.001	0.028 ± 0.011	0.009

Coefficients are based on linear mixed effects model controlling for bear ID

*NDVI* normalized difference vegetation index

## Discussion

In the Swedish boreal forest, berries are a critical autumn resource for bears to increase body mass prior to hibernation (Dahle et al. 1998; Stenset et al. 2016), but there is little information on how bears respond to spatial and temporal variation in food abundance during hyperphagia. We found that bears selected for areas of higher probability of berry occurrence and higher berry abundance than that found at random locations, providing support for our first hypothesis (H1). Surprisingly though, crowberry, a food resource previously identified as preferred by bears in years of low bilberry abundance (Stenset et al. 2016), occurred rarely at both random and foraging sample locations. Bilberries were the most available berry species and bears strongly selected for high-abundance bilberry patches, but not for high-abundance lingonberry patches (support for H2). This is in line with scat analyses, showing that bilberry is the most commonly used berry species (Stenset et al. 2016). We did not find that bears selected less for locations with a high abundance of bilberry and more for those with a high abundance of lingonberry towards the end of the study period to compensate for the temporal shift in abundance of the two berry species (contrary to H3). As expected (H4), variation in habitat type was an important determinant for bilberry and lingonberry occurrence, as well as abundance, and bears selected more strongly for very good berry patches in habitats with higher berry occurrence and abundance. Bears used lingonberries with a higher sugar content than those at random locations, but not better quality bilberries, providing partial support for H5. The specific factors determining the use of bilberry and lingonberry by bears therefore differed between the two species.

Omnivores are true generalists, and the diet composition of bears shows marked seasonality (Stenset et al. 2016). Berries ripen in autumn over vast areas of the Northern Hemisphere, and they provide important, yet seasonally restricted forage to bears. Berry-producing shrubs are the most common field shrubs in the Swedish boreal forests (Kardell 1979). However, we found that the predicted number of bilberries per square meter in random areas ranged between 26 berries/m^2^ at the beginning and 14 berries/m^2^ at the end of the sampling season. This was well below the reported bear foraging efficiency threshold of 44–50 berries/m^2^ found in different study systems (Pelchat and Ruff 1986; Welch et al. 1997). In our study system, bears selectively foraged on high-abundance bilberry locations with an estimated berry abundance that was three times higher (80 and 45 berries/m^2^ at the beginning and end of the sampling season, respectively) than at random locations and exceeded the minimum requirement of 44 berries at all times. We therefore assume that bears were able to maintain a high foraging efficiency when foraging on bilberries throughout our study period. Bilberry abundance in foraging plots dropped to the threshold of 44 berries/m^2^ at the end of our sampling period, suggesting that foraging for bilberries becomes inefficient later in the season (Welch et al. 1997). Higher-order selection by bears for patches with more berries than expected at random was hence crucial for finding a sufficient abundance of berries.

The quality, spatial occurrence, and abundance of each berry type differentially determined the selection of each. Lingonberry abundance increased throughout our sampling period and exceeded bilberry abundance in foraging plots on clearcuts after the end of August. Lingonberries were thus present in high numbers where bears foraged and constituted an important food resource. However, we did not find that bears actively selected for better-than-average lingonberry locations, which is in line with previous findings from our study area showing that bears used lingonberry less than expected (Stenset et al. 2016). We also did not find that bears used lingonberries disproportionally more when they became more available later in the season or bilberries disproportionally less when they became less available, even though their relative availabilities changed over time. Earlier studies in our study system demonstrated that bears readily use crowberries during autumn, in particular when bilberry crops are poor (Stenset et al. 2016). Contrary to these findings, but in line with our expectations, crowberries were least available and least used by bears, up to the point that reliable statistical predictions were unfeasible.

Bilberry and lingonberry not only differ in their time of ripening but also in their energetic properties and placement on the berry plant. Bilberries occurred in lower densities, were more evenly distributed on the plants, and were about a third heavier than lingonberries, which grew in dense clusters close to the foliage of the plant. Further, berry-producing bilberry shrubs were a third taller than berry-producing lingonberry shrubs. The differences in presentation suggest that bite sizes should be bigger and bite rate lower for lingonberries than for bilberries, but the structural properties and taller shrub height of bilberry may facilitate access to the berries for foraging bears. Coogan et al. (2014) compiled an in-depth review of the macronutrient (protein, lipid, carbohydrate) composition of several North American bear foods and reported that common blueberry (*Vaccinium myrtilloides*) and lingonberry (*V. vitis-idaea*) did not differ noticeably in total metabolizable energy content, nor in the composition of metabolizable energy coming from the different macronutrients (Table 1). From an energetic and nutritive standpoint, both species should therefore be approximately equal as food resources, despite the fact that ripe lingonberries in our study contained higher concentrations of sugar (TSS) than ripe bilberries. As lingonberries are about a third smaller than bilberries, bears would also need to ingest a third more berries to achieve the same energy gain, which might impose extra time costs of foraging. We conclude that bears in our study area concentrated foraging efforts on bilberry primarily because of their greater availability compared to lingonberry (Kardell 1979), but that they might forage on lingonberry opportunistically, especially later in the season. In years of low bilberry abundance, it should be profitable for bears to be plastic in their food search.

Land use, particularly forestry, can have a major influence on the distribution and abundance of berries. Kardell (1979) and Atlegrim and Sjöberg (1996) reported that clearcutting may reduce bilberry plant coverage by up to 70 %. Random sampling in our study confirmed that bilberry occurrence was higher in mature forests than on clearcuts. Bears responded to this by showing a higher selectivity for bilberry occurrence on clearcuts, up to the point that we found bilberries in almost 100 % of the foraging plots. Dense young forests with little light incidence are not productive bilberry habitats, because plant competition for light is high and berry shrubs consequently do not produce many fruits (Kardell and Eriksson 2011). Bears did not find areas of higher-than-average berry abundance in young forests. Lingonberries respond positively to the enhanced light conditions on clearcuts, and both berry occurrence and production may increase (Kardell 1980; Kardell and Eriksson 2011). In line with this, lingonberries occurred most often and reached the highest abundances on clearcuts in our study. Bilberry coverage requires about 55 years to fully recover from the impacts of clearcutting and reach the coverage found in mature forests (Kardell and Eriksson 2011). Short harvest rotation times and denser forest stands that enhance economic income (Linder and Östlund 1998) might negatively affect growing conditions for bilberries in the Swedish boreal forest. Swenson et al. (1999) have shown that 42 % of the forest in our study area is younger than 35 years, and thus low-productive berry habitat. Bergstedt and Milberg (2001) and Atlegrim and Sjöberg (1996) both showed that less intense logging techniques still reduce bilberry coverage, but less so than conventional clearcutting. We conclude that commercial forestry shapes the distribution and abundance of food for bears and consequently affects bear foraging patterns.

## Conclusion

Food availability during the hyperphagic phase in autumn is a prerequisite for successful winter hibernation and reproduction in bears (Rogers 1976; Stringham 1990). Further, lifetime reproductive success is affected by body condition as a subadult, and thus food availability experienced at that age (Zedrosser et al. 2013). Based on our findings that bears in central Sweden foraged primarily on bilberries, we suggest that a year with particularly low bilberry production potentially will have negative repercussions on reproductive success. Scandinavian brown bears used patches of above-average bilberry abundance, and we demonstrated that the selection for resource-rich patches was important for finding sufficiently high abundances of berries. We encourage further research on how animals navigate in human-altered landscapes, as we also found a major effect of land use on berry production. Brown bears are adapted to a wide range of environmental conditions and food types, but it remains to be seen how well local populations can respond to changes in the availability of food types.

## Electronic supplementary material

Below is the link to the electronic supplementary material.

ESM 1 (DOCX 1590 kb)

ESM 2 (DOCX 16 kb)
