# Supplementary material for: Bears and berries: species-specific selective foraging on a patchily distributed food resource in a human-altered landscape
Source: Behav Ecol Sociobiol. 2016 Mar 31;70:831–42. doi: 10.1007/s00265-016-2106-2 (PMC4859851; doi:10.1007/s00265-016-2106-2)
Supplement: Supplementary file 1 — (DOCX 1590 kb) [file 265_2016_2106_MOESM1_ESM.docx]

**Title:** Bears and berries: species-specific selective foraging on a patchily distributed food resource in a human-altered landscape.

**Journal:** Behavioral Ecology and Sociobiology

**Authors:** Anne G. Hertel^*1^, Sam M. J. G Steyaert^1^, Andreas Zedrosser^2,3^, Atle Mysterud^4^, Hanna K. Lodberg-Holm^1^, Henriette Wathne Gelink^1^, Jonas Kindberg^5^, Jon E. Swenson^1,6^

**Author Affiliations:** ^1^Norwegian University of Life Sciences, Department of Ecology and Natural Resource Management, 1430 Ås, Norway, ^2^Telemark University College, Department of Environmental and Health Sciences, 3901 Porsgrunn, Norway, ^3^University for Natural Resources and Life Sciences, Institute for Wildlife Biology and Game Management, 1180 Vienna, Austria , ^4^Centre for Ecological and Evolutionary Synthesis (CEES), Department of Biosciences, University of Oslo, NO-0316 Oslo, Norway, ^5^Swedish University of Agricultural Sciences, 90183 Umeå, Sweden, ^6^Norwegian Institute for Nature Research, 7485 Trondheim, Norway

**Corresponding Author E-mail**: anne.hertel@nmbu.no


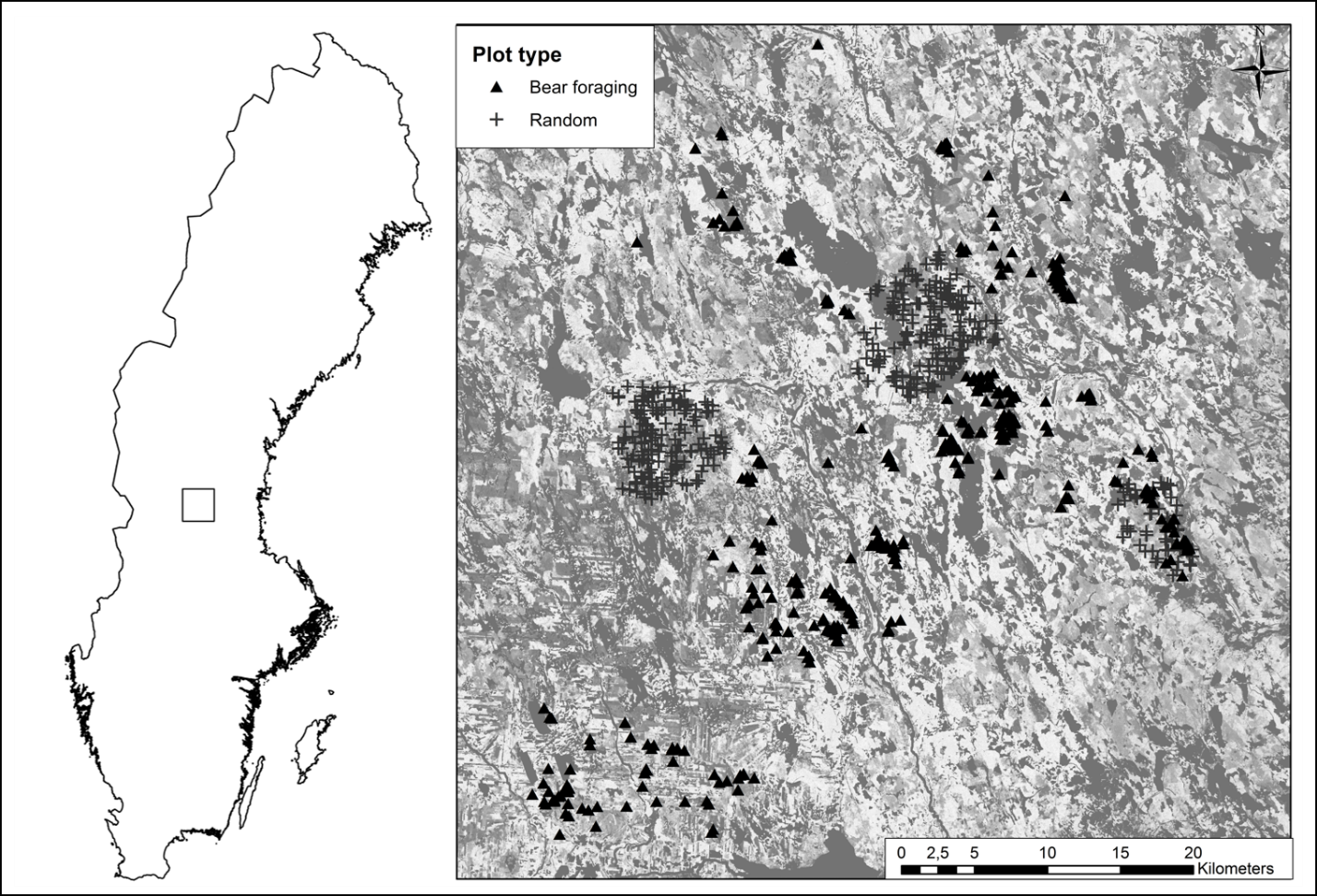


**Electronic supplemental material 1:** Location and extent of the study area in south-central Sweden, showing plot locations: Brown bear foraging plots are marked with filled triangles, random plots with crosses.
